# Toll-like receptor 4 agonist, lipopolysaccharide, increases the expression levels of cytokines and chemokines in human peripheral blood mononuclear cells

**DOI:** 10.3892/etm.2014.2025

**Published:** 2014-10-15

**Authors:** DAN PU, WEI WANG

**Affiliations:** 1 Clinical Skills Training Center, West China Hospital, Sichuan University, Chengdu, Sichuan 610041, P.R. China; 2 Department of Pathology, West China Second University Hospital, Sichuan University, Chengdu, Sichuan 610041, P.R. China

**Keywords:** Toll-like receptor 4, lipopolysaccharide, peripheral blood mononuclear cells, cytokines, chemokines, kinases, antibody chip array

## Abstract

Toll-like receptors (TLRs) are members of the pattern recognition receptor family and are essential in the innate immune response. In total, 11 TLRs exist in humans, which are expressed in a variety of cells, including peripheral blood cells. TLR4 plays a significant role in the defense against gram-negative pathogens by recognizing the lipopolysaccharide (LPS) molecules in these bacteria. The aim of the present study was to detect the expression level variation of a number of major immune molecules in peripheral blood mononuclear cells (PBMCs) stimulated by LPS, in order to identify candidate genes involved in the biological functions mediated by TLR4. Reverse transcription quantitative polymerase chain reaction (RT-qPCR) analysis and an antibody chip were performed in the current study. The RT-qPCR results revealed a marked enhancement in the expression levels of various molecules, including cytokines, chemokines, growth factors and protein kinases. In addition, the antibody chip identified the increased secretion of crucial proinflammatory molecules in the supernatants collected from LPS-treated PBMCs. In conclusion, a large number of molecules were found to be involved in TLR4-mediated functions.

## Introduction

Innate immunity is a major part of the immune system, playing a significant role in the acute inflammation induced by microbial infection or tissue damage (1). Innate immune cells, including monocytes, dendritic cells and T cells, can be recognized by their germ line-encoded pattern recognition receptors (PRRs), which are responsible for sensing the structure of microbial species (2). PRRs have also been shown to recognize endogenous ligands released from damaged cells and tissues (3).

The Toll-like receptor (TLR) family is a well-characterized PRR family that is responsible for recognizing invading pathogens (4). TLR stimulation initiates a signal transduction pathway via the adaptor protein, MyD88, which induces the expression of proinflammatory cytokines, such as interleukin (IL)-6, IL-8 and tumor necrosis factor (TNF)-α (5). In total, 11 members constitute the human TLR family. Among them, TLR4 is a transmembrane protein specialized in the recognition of lipopolysaccharide (LPS), which is a component of gram-negative bacteria (6).

A previous study has demonstrated that the activation of TLR4 induces the expression of numerous proinflammatory molecules, which are essential in shaping the immune status of immune cells (7). However, the expression level variation of immune molecules within immune cells upon TLR4 stimulation remains unclear. In the current study, peripheral blood mononuclear cells (PBMCs) were isolated from healthy volunteers and stimulated by the TLR4 agonist, LPS, while the expression levels of various cytokines, chemokines, growth factors and kinases were screened. The aim of the present study was to confirm which types of immune molecules are involved in the activation of the TLR4 signaling pathway.

## Materials and methods

### Isolation and stimulation of PBMCs

Heparinized venous blood samples were isolated from three healthy male volunteers (aged 25–28 years) and PBMCs were separated by density separation over Ficoll-Hypaque. After washing twice with phosphate buffered-saline, the PBMCs were plated into 24-well plates with a total number of 2×10^6^ cells/well. LPS was added to the PBMCs at a concentration of 100 ng/ml. Supernatants were collected at 4 h following stimulation for use in the antibody chip. The present study was approved by the Ethics Committee of West China Hospital, Sichuan University (Sichuan, China) and written informed consent was obtained from the patients.

### RNA extraction and cDNA synthesis

This procedure was performed as previously described (8). In brief, the total RNA from the PBMCs was extracted using a RNeasy mini kit (74104; Qiagen, Hilden, Germany) and quantified using a NanoDrop 3300 Fluorospectrometer (Thermo Fisher Scientific, Wilmington, DE, USA). cDNA was synthesized using a ReverTra Ace qPCR kit (FSQ-101; Toyobo, Kagoshima, Japan) and the reverse transcription (RT) conditions were as follows: 65°C for 5 min, 37°C for 15 min and 98°C for 5 min.

### Quantification polymerase chain reaction (PCR)

This procedure was performed as previously described (8). In brief, quantitative PCR (qPCR) was performed to amplify the synthesized cDNA using the RealMaster Mix Reagent (SYBR Green; FP202; Tiangen Biotech Co., Ltd., Beijing, China). An iCycler iQTM Optical Module (Beckman Coulter, Fullerton, CA, USA) was used for RT-qPCR under the following conditions: One cycle at 95°C for 30 sec, 40 cycles at 95°C for 30 sec, 58°C for 30 sec and 72°C for 30 sec, followed by a melt curve between 55 and 95°C in 0.5°C-increments and 10-sec intervals. All the tests were performed in triplicate and the primers used are shown in Table I.

### Antibody chip

The supernatants collected from the TLR4-stimulated and unstimulated PBMCs were arrayed for molecule secretion using the RayBio^®^ Human Antibody Array C-Series 1000 (RayBiotech, Inc., Norcross, GA, USA), according to the manufacturer’s instructions. Blots were analyzed using ImageJ software (National Institutes of Health, Bethesda, MD, USA).

### Statistical analysis

Data analysis was performed using the Bio-Rad iQ5 software (Bio-Rad Laboratories, Hercules, CA, USA), with glyceraldehyde 3-phosphate dehydrogenase as the internal control and normal PBMCs as the negative control. The results are expressed as the mean ± standard error of the mean and were analyzed using SPSS 16.0 software (SPSS, Inc., Chicago, IL, USA). P<0.05 and P<0.001 were considered to indicate a statistically significant difference when compared with the control group. The figures were obtained using GraphPad Prism 5 software (GraphPad Software, Inc., La Jolla, CA, USA).

## Results

### TLR4 agonist increases the expression levels of cytokines and chemokines

RT-qPCR was performed to screen the expression levels of a number of cytokines and chemokines, in order to identify candidate genes responsible for the LPS-mediated changes in PBMCs. The expression level variation of a number of these genes may significantly affect the biological function of the PBMCs, induced by microenvironment or pathogen stimulation.

**Figure 1:**
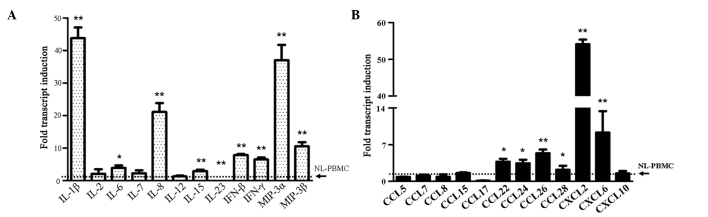
Gene expression level variation in (A) cytokines and (B) chemokines, as detected by quantitative reverse transcription polymerase chain reaction. ^*^P<0.05 and ^**^P<0.001, vs. control group. IL, interleukin; IFN, interferon; MIP, macrophage inflammatory protein; CCL, chemokine (C-C motif) ligand; CXCL, chemokine (C-X-C motif) ligand; NL-PBMC, normal peripheral blood mononuclear cell.

With regard to cytokine detection, the TLR4 agonist, LPS, was found to significantly increase the expression levels of several major cytokines (P<0.001), including IL-1β, IL-8, IL-15, interferon (IFN)-β, IFN-γ, macrophage inflammatory protein (MIP)-3α and MIP-β. In addition, LPS slightly increased the expression level of IL-6 (P<0.05). IL-23 was the only downregulated cytokine detected (P<0.001; Fig. 1A). During the chemokine assay, only the chemokine (C-C motif) ligand (CCL) 26, chemokine (C-X-C motif) ligand (CXCL) 2 and CXCL6 were found to be evidently enhanced upon LPS stimulation (P<0.001), although CCL22, CCL24 and CCL28 were also found to be significantly different compared with the controls (P<0.05; Fig. 1B).

### LPS stimulation enhances the expression levels of growth factors

**Figure 2:**
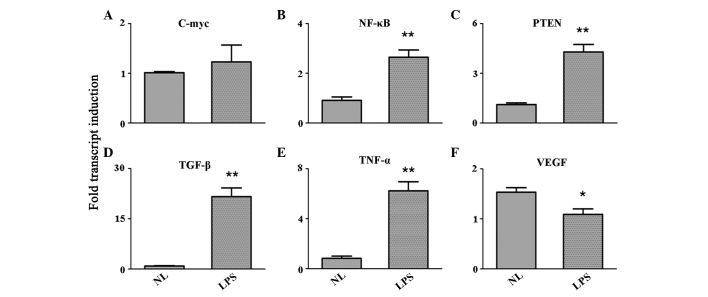
Expression level variation of the growth factors, (A) c-Myc, (B) NF-κB, (C) PTEN, (D) TGF-β, (E) TNF-α and (F) VEGF, as detected by quantitative reverse transcription polymerase chain reaction. ^*^P<0.05 and ^**^P<0.001, vs. control group. NF, nuclear factor; PTEN, phosphatase and tensin homolog; TGF, transforming growth factor; TNF, tumor necrosis factor; VEGF, vascular endothelial growth factor; NL, normal PBMCs; LPS, lipopolysaccharide-treated PBMCs; PBMCs, peripheral blood mononuclear cells.

Growth factors were screened to obtain their expression levels following LPS stimulation. The results indicated an evident upregulation of nuclear factor (NF)-κB, phosphatase and tensin homolog (PTEN), transforming growth factor (TGF)-β and TNF-α in the PBMCs activated by the TLR ligand (P<0.001). The expression of c-Myc remained unchanged, while the expression of vascular endothelial growth factor (VEGF) was inhibited (P<0.05; Fig. 2).

### Activation of the TLR4 signaling pathway has no evident effect on the expression levels of kinases

**Figure 3:**
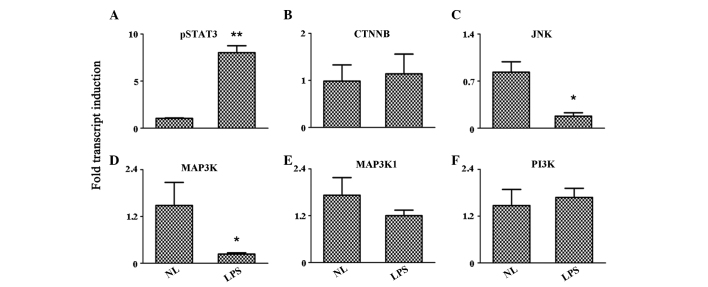
Expression level variation of the protein kinases, (A) pSTAT3, (B) CTNNB, (C) JNK, (D) MAP3K, (E) MAP3K1 and (F) P13K, as detected by quantitative reverse transcription polymerase chain reaction. ^*^P<0.05 and ^**^P<0.001, vs. control group. pSTAT3, phosphorylated signal transducer and activator of transcription 3; CTNNB, cadherin-associated protein β; JNK, c-Jun N-terminal kinase; MAP3K, mitogen-activated protein kinase kinase kinase; PI3K, phosphatidylinositol-4,5-bisphosphate 3-kinase; NL, normal PBMCs; LPS, lipopolysaccharide-treated PBMCs; PBMCs, peripheral blood mononuclear cells.

TLR4 agonist stimulation performed on PBMCs may induce the activation of protein kinases. Therefore, a number of protein kinase signaling pathways were analyzed in the study. The results indicated that the expression of phosphorylated signal transducer and activator of transcription 3 (pSTAT3) was significantly enhanced in PBMCs following LPS stimulation (P<0.001). However, cadherin-associated protein β (CTNNB), mitogen-activated protein kinase kinase kinase 1 (MAP3K1) and phosphatidylinositol-4,5-bisphosphate 3-kinase (PI3K) expression levels remained unchanged during the detection. Notably, the expression levels of two important kinases, c-Jun N-terminal kinase (JNK) and MAP3K, were inhibited following LPS treatment (P<0.05; Fig. 3).

### LPS induces the secretion of proinflammatory molecules

**Figure 4:**
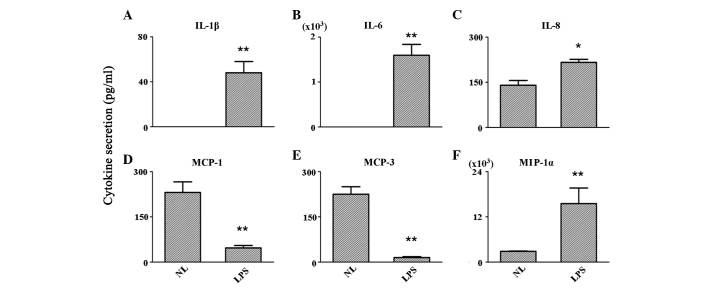
Secretion levels of (A) IL-1β, (B) IL-6, (C) IL-8, (D) MCP-1, (E) MCP-3 and (F) MIP-1α in the supernatant. ^*^P<0.05 and ^**^P<0.001, vs. control group. IL, interleukin; MCP, monocyte chemoattractant protein; MIP, macrophage inflammatory protein; NL, normal control PBMCs; LPS, lipopolysaccharide-treated PBMCs; PBMCs, peripheral blood mononuclear cells.

Supernatants collected from the LPS-treated and untreated PBMCs were analyzed to determine the secretion levels of major immune molecules using an antibody chip. A total of 20 proteins, including α-fetoprotein, albumin, E-selectin, intercellular adhesion molecule-1, IFN-α, IFN-γ, IL-10, IL-12, IL-18, IL-1β, IL-4, IL-5, IL-6, IL-8, monocyte chemoattractant protein (MCP)-1, MCP-3, MIP-1α, Notch-1, TGF-β and VEGF, were selected for analysis. The results revealed that only six of the aforementioned proteins were affected by TLR4 activation (Fig 4). Among these, the secretion levels of IL-1β, IL-6 and MIP-1α were significantly enhanced (P<0.001), while IL-8 secretion was moderately increased (P<0.05). In addition, the secretion levels of MCP-1 and MCP-3 were inhibited.

## Discussion

Vertebrates have evolved systems of immune defense to eliminate invading pathogens. The innate immune system is the first line of the host defense against microorganisms and recognizes the conserved components of pathogens via pattern recognition receptors (PRRs) (9). Among the 11 human TLRs, TLR3, TLR7, TLR8 and TLR9 are expressed on the surface of endosomes, while TLR1, TLR2, TLR4, TLR5, TLR6, TLR10 and TLR11 are located on the cellular surface (10). TLR4 plays an important role in the protection against fungi by recognizing LPS components. Stimulation with a TLR4 agonist induces proinflammatory signals, as well as the production of type I IFNs (11).

In the present study, PBMCs were isolated from healthy volunteers and stimulated by the TLR4 agonist, LPS. At 4 h after the treatment, the supernatant and cellular RNA were isolated and assayed using an antibody chip and RT-qPCR, respectively. Using these methods, the immune molecules involved in the immunological signature of PBMCs can be identified and the TLR4 signaling pathway can be further understood. The molecules selected in the present study exhibit a number of important biological functions *in vivo*. In addition to increasing the expression levels of IL-8 and IL-6 (12), TLR4 agonist stimulation was found to influence numerous immunomodulatory factors. Among the cytokines examined, the expression levels of IL-1β, IL-6, IL-8, IL-15, IFN-β, IFN-γ, MIP-3α and MIP-3β increased markedly. During chemokine detection, the expression levels of CCL22, CCL24, CCL26, CXCL2 and CXCL6 were upregulated upon LPS stimulation. In addition, the expression levels of several growth factors, including NF-κB, PTEN, TNF-α and TGF-β, were enhanced. Detecting the expression levels of protein kinases, which play important roles in TLR-mediated biological functions, was important in this study. The expression level of pSTAT3 was found to increase in LPS-treated PBMCs, while the expression levels of CTNNB, MAP3K1 and PI3K remained unchanged in the treated and untreated groups. However, the expression of JNK was inhibited following LPS treatment. These results indicated that the activation of TLR4 had varying effects on the different kinase pathways. The cytokine levels were detected in the supernatants of LPS-treated PBMCs, and the secretion levels of IL-1β, IL-6, IL-8 and MIP-1α were found to be enhanced, indicating the important role of TLR4 in shaping the immune status of PBMCs.

Compared with previous studies, the present study analyzed a greater number of immune molecules that may be important in TLR-related functions. However, a limitation of the study was that the variation in the expression levels of immune molecules was only detected at a gene level. Future studies should analyze the association between these candidate immune molecules and TLR-mediated functions and should investigate the MEprotein expression levels.

## Figures and Tables

**Table I:** Oligonucleotides used in quantitative polymerase chain reaction analysis.

Gene	Forward primer	Reverse primer	GenBank number
CCL5	GACACCACACCCTGCTGCT	TACTCCTTGATGTGGGCACG	NM_002985
CCL7	AGCAGAGGCTGGAGAGCTACA	GGGTCAGCACAGATCTCCTTGT	NM_006273
CCL8	GTTTCTGCAGCGCTTCTGTG	TGGCTGAGCAAGTCCCTGA	Y10802
CCL15	CCTCTCCTGCCTCATGCTTATT	CTCTGTCTCTGCATCATTTGTGAA	U58914
CCL17	CCATCGTTTTTGTAACTGTGCAG	TGCATTCTTCACTCTCTTGTTGTTG	NM_002987
CCL22	TGCGCGTGGTGAAACACT	GGTTAGCAACACCACGCCA	NM_002990
CCL24	AGCCTTCTGTTCCTTGGTGTCT	GGGAGAGGGTATGACCACAGAG	NM_002991
CCL26	CCAAGACCTGCTGCTTCCAA	GAATTCATAGCTTCGCACCCA	NM_006072
CCL28	CTCGCCATCGTGGCCTT	GCAATGGGAAGTATGGCTTCTG	AF220210
c-Myc	CAAGACTCCAGCGCCTTCTC	GTTGAGTAACGAGCTGACCCC	AM393287
CTNNB	CATCGTGAGGGCTTACTGGC	GAGCAAGGCAACCATTTTCTG	XM_006712984
CXCL2	AGGTGAAGTCCCCCGGAC	GCCCATTCTTGAGTGTGGCT	NM_002089
CXCL6	GCTGAGAGTAAACCCCAAAACG	GGAGCACTGCGGGCC	NM_002993
GAPDH	GAAGGTGAAGGTCGGAGTC	GAAGATGGTGATGGGATTTC	J04038
IFN-β	CAGCAATTTTCAGTGTCAGAAGCT	TCATCCTGTCCTTGAGGCAGT	M28622
IFN-γ	CCAACGCAAAGCAATACATGA	CGCTTCCCTGTTTTAGCTGC	J00219
IL-1β	ACGAATCTCCGACCACCACT	CCATGGCCACAACAACTGAC	M15330
IL-2	CAAGAATCCCAAACTCACCAGG	GACACTGAAGATGTTTCAGTTCTGT	J00264
IL-6	GACCCAACCACAAATGCCA	GTCATGTCCTGCAGCCACTG	M14584
IL-7	ACCAGTAGAAGACAATTGCATC	CCAGGTTTTCATCATCTTCAGCT	NM_001199888
IL-8	CTGGCCGTGGCTCTCTTG	CCTTGGCAAAACTGCACCTT	NM_000584
IL-12	CGGTCATCTGCCGCAAA	CAAGATGAGCTATAGTAGCGGTCCT	M65272
IL-15	GACCCCACCAAAGCTGGAC	TCACAGTGCTGCTGTCTGCTG	M90391
IL-23	GAAGGCTCCGCTCTGCAAT	TCTGGGTCTTCTCGATGGCA	L06801
IP-10	TGAAATTATTCCTGCAAGCCAA	CAGACATCTCTTCTCACCCTTCTTT	NM_001565
JNK	GCTAATTCTGTACCAATGTC	GAAGAGTGCACGTCAGGAAC	NM_139049
MAP3K	CCTGCTCGGTGCACGATGCTG	CTCTGTCTCTTCACGTGGCGG	NM_003954
MAP3K1	CTTTTAAGTCAGAAGTTGCTG	CTTCTCCATTTTCAACCTGC	AF042838
MIP-3α	TCCTGGCTGCTTTGATGTCA	TCAAAGTTGCTTGCTGCTTCTG	NM_004591
MIP-3β	GGCACCAATGATGCTGAAGA	GAAGTTCCTCACGATGTACCCAG	NM_006274
NF-κB	AGAGTGCTGGAGTTCAGGATA	AAGGTGGATGATTGCTAAGTGT	AJ271718
PTEN	ACCATAACCCACCACAGC	CAGTTCGTCCCTTTCCAG	NM_058074
PI3K	CCTGGGGGTTGGTGGCTGTTC	GTCTGGCTGGAATGATGCTATC	NM_006219
STAT3	CCTACAAAGGGGACCCCATTGTAC	CAGGGAATTTGACCAGCAACC	NM_213662
TGF-α	TATCGACATGGAGCTGGTGAAG	CAGCTTGGACAGGATCTGGC	X02812
TNF-β	GGTGCTTGTTCCTCAGCCTC	CAGGCAGAAGAGCGTGGTG	M10988
VEGF	GACTTGAGTTGGGAGGGGAA	GAGGCTCAGCGCCAGGGCTGGG	AF024710

CCL, chemokine (C-C motif) ligand; CTNNB, cadherin-associated protein β; CXCL, chemokine (C-X-C motif) ligand; GAPDH, glyceraldehyde 3-phosphate dehydrogenase; IFN, interferon; IL, interleukin; IP, interferon γ-induced protein; JNK, c-Jun N-terminal kinase; MAP3K, mitogen-activated protein kinase kinase kinase; MIP, macrophage inflammatory protein; NF, nuclear factor; PTEN, phosphatase and tensin homolog; PI3K, phosphatidylinositol-4,5-bisphosphate 3-kinase; STAT, signal transducer and activator of transcription; TGF, transforming growth factor; TNF, tumor necrosis factor; VEGF, vascular endothelial growth factor.
